# Dry season ecology of *Anopheles gambiae *complex mosquitoes at larval habitats in two traditionally semi-arid villages in Baringo, Kenya

**DOI:** 10.1186/1756-3305-4-25

**Published:** 2011-02-28

**Authors:** Albert O Mala, Lucy W Irungu, Josephat I Shililu, Ephantus J Muturi, Charles C Mbogo, Joseph K Njagi, John I Githure

**Affiliations:** 1 Human Health Division, International Centre of Insect Physiology and Ecology, P.O. Box 30772-00100, Nairobi, Kenya; 2 School of Biological Sciences, University of Nairobi, Nairobi, Kenya; 3 Illinois Natural History Survey, University of Illinois, IL, USA; 4 Kenya Medical Research Institute, Kenya; 5 Ministry of Health, Division of Malaria Control, Nairobi, Kenya

## Abstract

**Background:**

Pre-adult stages of malaria vectors in semi-arid areas are confronted with highly variable and challenging climatic conditions. The objective of this study was to determine which larval habitat types are most productive in terms of larval densities in the dry and wet seasons within semi-arid environments, and how vector species productivity is partitioned over time.

**Methods:**

Larval habitats were mapped and larvae sampled longitudinally using standard dipping techniques. Larvae were identified to species level morphologically using taxonomic keys and to sub-species by polymerase chain reaction (PCR) methods. Physical characteristics of larval habitats, including water depth, turbidity, and presence of floating and emergent vegetation were recorded. Water depth was measured using a metal ruler. Turbidity, pH, conductivity, dissolved oxygen, temperatures salinity and total dissolved solids (TDS) were measured in the field using the hand-held water chemistry meters.

**Results:**

Mean larval densities were higher in the dry season than during the wet season but the differences in density were not statistically significant (F = 0.04, df = 1, p = 0.8501). Significantly higher densities of larvae were collected in habitats that were shaded and holding turbid, temporary and still water. Presence of emergent or floating vegetation, habitat depth, habitat size and habitat distance to the nearest house did not significantly affect larval density in both villages. There was a weakly positive relationship between larval density and salinity (r = 0.19, p < 0.05), conductivity (r = 0.05, p = 0.45) and total dissolved solids (r = 0.17, p < 0.05). However, the relationship between water temperature and larval density was weakly negative (r = 0.15, p = 0.35). All statistical tests were significant at alpha = 0.05.

**Conclusion:**

Breeding of malaria vector mosquitoes in Baringo is driven by predominantly human-made and permanent breeding sites in which *Anopheles arabiensis *and *Anopheles funestus *breed at a low level throughout the year. Permanent water sources available during the dry season serve as inocula by providing "larval seed" to freshly formed rain-fed habitats during the rainy season. The highly localized and focal nature of breeding sites in these semi-desert environments provides a good opportunity for targeted larval control since the habitats are few, well-defined and easily traceable.

## Background

One usually does not associate malaria with a semi-arid biological environment. Common sense dictates that malaria-carrying mosquitoes that breed mainly in stagnant water would give water-scarce areas a wide berth. Contrary to this belief, most semi-arid complexes are currently hit by malaria epidemics as highlighted by reports on paediatric admissions in semi-arid districts in Kenya [1].

Several factors may be responsible for this state of affairs. Permanent water sources in dry lands provide potential vectors with water for most of the year, ensuring year-round low level malaria transmission. The hand of poverty has also been implicated. Populations in North- West and North-Eastern Kenya are poor, semi-nomadic communities with little acquired functional immunity to *Plasmodium falciparum *due to infrequent challenge by malaria [2]. This has ensured the disease remains life-threatening to all age groups in these areas. Malnutrition and public health policy bias could also be blamed for a dearth of information on dry land malaria entomology. In Kenya, for example, the 2001-2009 Kenya Government national malaria strategy [3] marginalized communities living in semi-arid areas because government public health technocrats assumed they were not exposed to malaria risk.

It is not too late to develop sustainable interventions that could bring malaria transmission in these areas under control. The unique ecological features found in arid areas make larval control an even more feasible tool than in high rainfall areas. This is because larval habitats in these ecosystems occur seasonally or are relatively limited and well defined [4]. If the focal sites where mosquitoes breed in semi-arid/arid environments and during the dry season can be identified and managed, then the reservoir of vector species that form "seed" at the onset of the rains would be eliminated [5]. We envision that countries lying within the semi-arid regions of Africa would have a more sustained approach to control of malaria vectors if the larval ecology of vector species resident in them is adequately understood. It is likely that the results of this study will shed an understanding on spatial and temporal heterogeneities experienced in malaria transmission in these regions.

## Methods

### Study site

**Figure 1:**
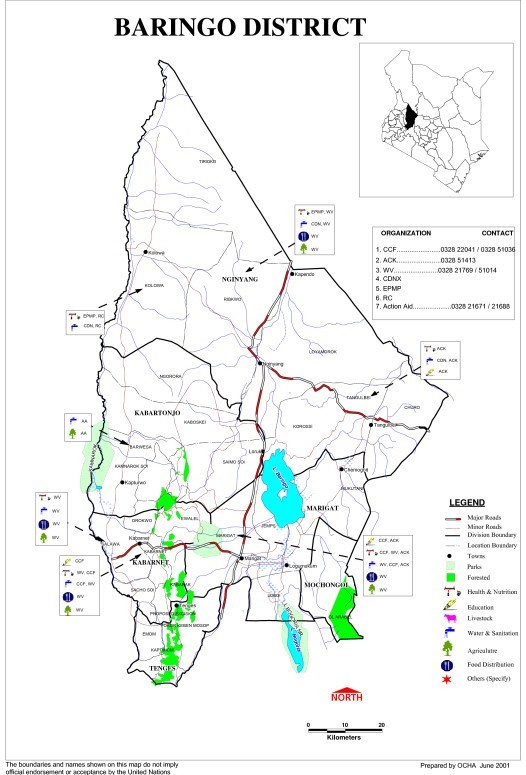
**Map of Baringo District showing the study area**.

The study was conducted in Kamarimar and Tirion villages in Marigat division of Baringo district in Kenya. The two villages are located approximately 20 km and 17 km respectively, away from Marigat town (Figure 1). The town is about 250 km north-west of Nairobi and is situated 0.45N and 36E. Accessibility and availability of known breeding sites are the factors that influenced site choice. The division is semi-arid with an average but unreliable annual rainfall of between 500 and 600 mm, coupled with high average temperature of above 32°C that results in elimination of temporary standing water in a matter of days. The average altitude of the study area is about 700 meters above the sea level and most of it is rangelands with pastoralism being the main activity. The main rainy season occurs between the months of March and June. The short rains come between October and December but in some years these are scanty or totally absent. There is usually a long dry period from October to February whenever the short rains fail, characterized by high temperatures and strong dusty winds, especially from January, with little rainfall. These harsh ecological conditions ensure only permanent water sources remain the foci of *Anopheles gambiae *s.l and *Anopheles funestus *breeding, which occurs in low numbers through out the year [6-8].

### Habitat census

All water bodies were located and mapped with geopositioning equipment (GPS) in July 2008. A total of 25 discrete habitats (14 and 11 in Kamarimar and Tirion respectively) were mapped and assigned numbers. Each habitat was sampled by visual inspection, dipper, and hand-picking with a pipette for preliminary classification by presence or absence of anopheline and/or culicine larvae. Distance of each water body to the nearest house was estimated from Geographic Information System (GIS) maps of the study area.

### Larval sampling

All potential breeding sites were sampled longitudinally using a standard mosquito dipper (350 mL) once weekly for a period of 22 months from July 2008 to April 2010. Ten dips were taken from each habitat. In small habitats where this was not practical, larvae were collected individually using plastic pipettes on a daily basis. Larvae were then transferred from the dipper by pipetting into a white collecting tray with clear water for categorization into different instar stages, followed by counting, morphological identification and recording [9]. The 3rd and 4th instar anophelines were identified morphologically using taxonomic keys of Gillies and De Meillon [10] and Gillies and Coetzee [11]. Larvae were reared and 500 randomly selected emerged *Anopheles gambiae s.l *adults identified to sub-species by polymerase chain reaction (PCR) methods [12].

### Water chemistry analysis

Physical characteristics of the larval habitats, including water depth, turbidity, presence of floating and/or emergent vegetation were recorded. Water depth was measured using a metal ruler. Turbidity, which was mainly caused by suspended organic matter, was measured through visual examination of water against a white background and categorized as either clear or turbid. A record of whether the habitat was wet or dry at the time of the visit was also taken. Water pH, conductivity, and temperature were measured using hand-held YSI 650 Multiparameter Display System (YSI Environmental, YSI Incorporated, Yellow Springs, OH). Salinity and TDS were measured in the field using the hand-held YSI EC 300 (YSI Environmental).

### Data analysis

Data analyses were performed using SAS version 9.1 for Windows (SPSS Inc., SAS Institute). Physical habitat characteristics such as habitat size, stability, and distance to the nearest house were categorized as dichotomous variables for analysis. The cut-offs for each variable was selected to maximize the number of habitats within each category using the methods of Mutuku and others [13]. Habitats were classified as large if their areas were greater than 5 m^2^. For stability, habitats were classified as stable if they were flooded for at least 18 days. For distance to the nearest house, habitats were classified as near if they were within 50 m of a human dwelling and far if they were greater than 50 m from a human dwelling. Variation in larval counts between villages and seasons was compared by Student *t*-test, and differences in larval counts among habitat types and months analyzed using one-way analysis of variance (ANOVA). Where significant differences were observed in ANOVA, the Tukey test was used to separate the means. Variation in diversity of habitat types between villages was compared using the Chi-square test. Pearson correlation analysis was used to assess the relationship between water chemistry covariates and larval counts in different habitat types and villages. Variation in larval densities and categories of habitat characteristics were analyzed using one-way analysis of variance (ANOVA). Larval counts were expressed as the number of larvae per 20 dips/7000 mL (350 mL × 20) because the number of larvae sampled was low. Statistical analyses was done using log-transformed (log10 n + 1) larval counts to normalize the data. Results were considered significant at *P *< 0.05.

## Results

### Habitat survey

**Figure 2:**
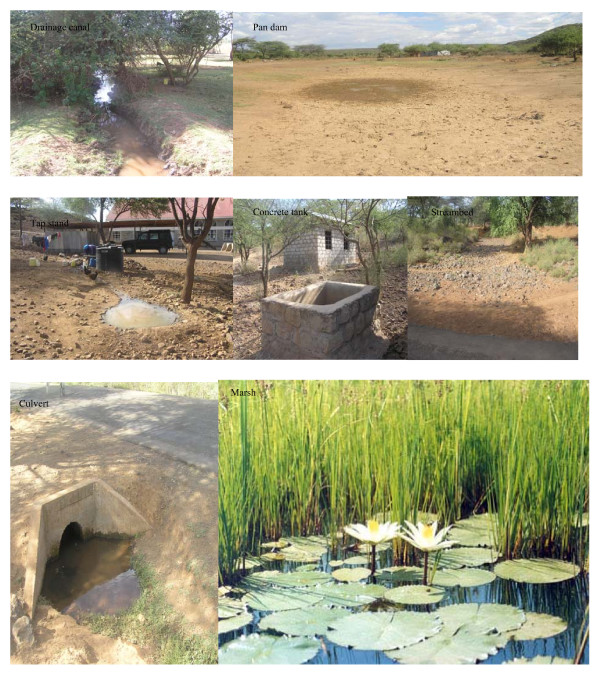
**Types of breeding sites in Baringo**.

A total of 25 discrete habitats were mapped and their mode of formation recorded (Figure 2). In Kamarimar, majorities of breeding sites (78.57%) were man-made in origin, 7.14% were livestock-associated, and the remainders were naturally occurring. In Tirion Village, 90.9% of all habitats were man-made and the remainder naturally occurring. Chances of sampling anopheline mosquito larvae were higher in marshes and canals in Kamarimar but highly heterogeneous in Tirion where a majority of habitat types were supportive to anopheline larval development (Table 1).

**Table 1:** Habitat types and mosquito larval prevalence in aquatic habitats from July 2008 to April 2010

Village	Habitat type	No. of times with Anopheline larvae only	No. of times with Culcine larvae only	No. of times with both Anopheline and Culcine larvae	No. of times without larvae	Total (%)
**Kamarimar**	Concrete tank	2	19	8	11	40 (11.1)
	Hoof print	0	0	0	4	4 (1.1)
	Marsh	7	3	7	10	27 (7.5)
	Canal	17	121	85	52	275 (75.6)
	Tyre tracks	0	0	0	3	3 (0.8)
	Stream beds	0	0	0	13	13 (3.6)
	**Total/mean**	**26**	**143**	**100**	**93**	
**Tirion**	Concrete tank	2	0	2	3	7 (1.8)
	Culvert	4	11	10	7	32 (8.2)
	Ditch	13	6	19	15	53 (13.6)
	Marsh	4	2	10	11	27 (6.9)
	Canal	1	3	2	145	151 (38.8)
	Pandam	27	7	28	57	119 (30.5)

	**Total/mean**	**51**	**29**	**72**	**238**	

### Larval abundance and habitat diversity

**Figure 3:**
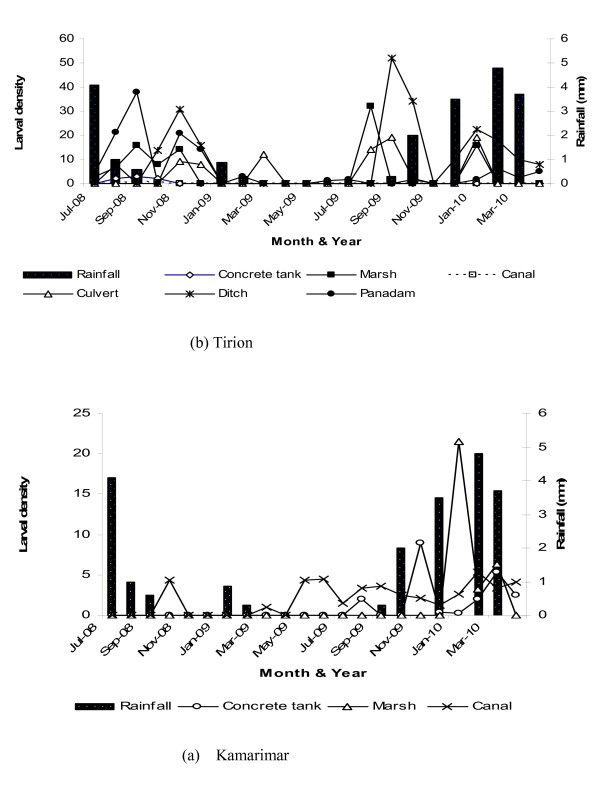
**Seasonal contribution of different habitat types to Anopheline larval production (all stages) over the 22-month sampling period in two villages**.

A total of 590 larvae (371 early instars, 219 late instars) were collected in Kamarimar and 1249 (1000 early instars, 294 late instars) in Tirion. (Table 2). Habitat support for larval development varied in the two villages. In Kamarimar, 26 habitats had Anopheline larvae only and were visited 363 times compared to 51 in Tirion which were visited 389 times resulting in an overall tally of 752 longitudinal samples in 22 months (Table 2). The relative abundance of early (t = 3.87, df = 1, *P *< 0.0001) and late instars (t = 5.91, df = 1, *P *< 0.0001) were significantly higher in Tirion than Kamarimar. Larval densities for early and late instars were two-fold and five-fold respectively, higher in Tirion than Kamarimar. The temporal dynamics of different habitat types with regard to larval presence and productivity is shown in Figure 3.

**Table 2:** Relative abundance of anopheline larvae collected from different habitat types and the proportion of aquatic habitats positive for Anopheline larvae

Village	Habitat type	No. of habitats	No. of samples taken	Percentage positive Anopheline larvae	Counts of early instars/10 dips	Counts of late instars/10 dips
**Kamarimar**	Concrete Tank	2	36	25	24	27
	Hoof print	1	4	0	0	0
	Marsh	3	27	51.85	100	26
	Canal	6	280	37.09	247	166
	Tyre tracks	1	3	0	0	0
	Stream beds	1	13	0	0	0
	**Total/mean**	**14**	**363**		**371**	**219**
**Tirion**	Concrete tank	1	7	57.14	7	0
	Culvert	1	33	43.75	80	36
	Ditch	2	53	60.38	310	126
	Marsh	1	27	51.85	106	13
	Canal	3	150	1.99	7	0
	Pandam	3	119	40.34	490	119

	**Total/mean**	**11**	**389**		**1000**	**294**

Six distinct habitat types were identified in each village (Table 1). Canal, marsh, and concrete tank habitats constituted most of the samples in Kamarimar, while pan dam, ditch, marsh, and culvert habitats constituted most of the samples in Tirion. Results of ANOVA and Turkey's honestly significant differences test showed counts of late instars of anopheline larvae in Tirion were significantly higher in pan dams, canals, concrete tanks and in ditches compared with the other habitat types (F = 5.82, df = p < 0.001). Similar analyses in Kamarimar revealed significantly higher larval counts in marshes, canals and concrete tanks than in the other habitat types (F = 5.82, df = 2, p < 0.001). However, in relation to long-term contribution to larval productivity, canals were more important because they had water available for anopheline larval development long after most of the other habitats had dried up. They were therefore sampled more times for mosquito larvae compared with other habitat types (Table 2).

### Larval abundance and season

The importance of habitats in larval production was dependent upon the month of collection and village (Figure 3). Some habitats were important in one village in a particular month (F = 3.80, df = 20, *P *< 0.0001), but were either absent or less important in relation to habitats in the other village. In Kamarimar, concrete tanks showed seasonal variability and supported larval development only in the wet months of August 2009 all through to April 2010 (Figure 3a). Marshes supported larval development for only three wet season months (January to March) out of 22 months. Canals were the most stable breeding sites and supported year- round larval production regardless of season. They supported larval survival through the long dry season that extended from March to August in the year 2009. In Tirion, canals supported minimal larval development in September, October and November in the year 2008 only, with no evident seasonal variation (Figure 3b). Larval production was largely varied with season in pan dams, culverts, concrete tanks, ditches and marshes. These habitat types supported larval development through most the wet months of September, December and November in the year 2008, in January, February, July, November and December in the year 2009 and in April in the year 2010. Overall, there were no significant differences in larval densities among different months in both villages. Mean larval densities were higher in the dry season (0.61 ± 0.97) than the wet season (0.51 ± 0.88) but the differences in density were not statistically significant (F = 0.04, df = 1, p = 0.8501).

### Species composition and abundance of anopheline larvae

Some 41.33% (n = 212) of late stage anopheline larvae were positively identified. *Anopheles gambiae *s.l constituted 55.04% and *Anopheles pharoensis *46.7% in Kamarimar. In Tirion, 44.30% of anophelines were *Anopheles gambiae *s.l while 50.63% were *Anopheles pharoensis *(Table 3). *Anopheles coustani *and *Anopheles funestus *were available in Tirion Village only. PCR results showed all 500 *Anopheles gambaie *complex mosquitoes were *Anopheles arabiensis*, making this sub-species by far the most abundant anopheline mosquito in both villages.

**Table 3:** Distribution of anopheline larval species in different larval habitat types

Village	Anopheles Species	Marsh	Canal	Concrete tank	Hoof print	Tyre tracks	Stream beds	Ditch	Pandam	Culvert	Total (%)
**Kamarimar**	*Anopheles gambiae*	18	46	7	0	0	0	*	*	*	71(55.04)
	*Anopheles coustani*	0	0	0	0	0	0	*	*	*	0(0.0)
	*Anopheles funestus*	0	0	0	0	0	0	*	*	*	0(0.0)
	*Anopheles pharoensis*	31	26	1	0	0	0	*	*	*	58(44.96)
	Total	49	72	8	0	0	0	*	*	*	129
**Tirion**	*Anopheles gambiae*	5	0	0	*	*	*	21	9	0	35(44.30)
	*Anopheles coustani*	3	0	0	*	*	*	2	0	0	5(6.32)
	*Anopheles funestus*	0	0	0	*	*	*	0	2	0	2(2.53)
	*Anopheles pharoensis*	5	0	0	*	*	*	18	17	1	40(50.63)

	Total	13	0	0	*	*	*	39	26	1	83

Key: *...indicates that habitat type was absent.

Overall, *Anopheles gambiae *and *Anopheles pharoensis *jointly accounted for 96.7% of all mosquitoes and were represented in all habitat types except in hoof prints, tyre tracks and stream beds in Kamarimar. In Tirion, these species were absent in canals and concrete tanks but present in marshes ditches, pan dams and culverts.

### Factors associated with larval development in aquatic habitats

Significantly higher densities of larvae were collected in shaded habitats holding turbid, temporary and still water (Table 4). Presence of emergent or floating vegetation, depth, size and distance to the nearest house did not significantly affect larval density among the two villages. There was a weakly positive relationship between larval density and salinity (r = 0.19, p < 0.05), conductivity (r = 0.05, p = 0.45) and total dissolved solids (r = 0.17, p < 0.05). However, the relationship between water temperature and larval density was weakly negative (r = 0.15, p = 0.35).

**Table 4:** Larval habitat characteristics and mean densities of *Anopheles gambiae *larvae collected

Habitat characteristics		Mean ± SD	F	**Sig**.
Shade intensity	Light	1.54 ± 3.74	4.70	0.036
	Shade	4.08 ± 9.12		
Turbidity	Clear	1.42 ± 3.53	3.79	0.029
	Turbid	5.32 ± 9.72		
Water depth	Deep	2.09 ± 5.95	0.59	0.447
	Shallow	3.42 ± 7.27		
Vegetation cover	Floating	4.03 ± 8.88	2.53	0.089
	Emergent	1.73 ± 4.28		
	Floating+ Emergent	3.48 ± 9.70		
Permanence	Permanent	1.54 ± 3.74	4.70	0.036
	Temporary	4.08 ± 9.12		
Size	Large	2.21 ± 6.01	0.62	0.43
	Small	3.42 ± 7.52		
Water current	Moving	1.30 ± 3.14	6.70	0.0132
	Still	3.97 ± 8.74		
Distance to nearest house	Near	2.44 ± 6.37	0.14	0.714

	Far	3.19 ± 6.27		

## Discussion

The results of this study show a highly restricted structure in terms of habitat type and larval species abundance and diversity; with permanent and localized water sources being the drivers of year-round low-level larval production. Lack of diversity in habitat types had a marked effect on *Anopheles *species diversity. Tirion, which had significantly more diverse habitat types, had a richer *Anopheles *mosquito fauna than Kamarimar, which had fewer habitat types. Previous findings have reported a close association between larval habitat diversity and mosquito fauna [14,15]. An alternative explanation for lack of larval species diversity could be found in the fact that a permanent swamp was the sole water source in the two villages. The villages are joined by a major irrigation canal that drains from this swamp and provides residents with water for domestic use. This created a scenario in which sampling was done in two different habitat types (marshes and drainage canals) but was essentially done from the same water body, especially during the dry season when only these permanent water sources supplied larvae.

Human activities and climatic changes tremendously influenced larval breeding. Over 90% of all habitats in both villages were by-products of human activities, attesting to the human-dependent ecology of Afrotropical *Anopheles *[16]. By 4 weeks after the end of the rainy season, most water bodies had dried up and few mosquito larvae could be found. As a result, the number of adult mosquitoes collected in surrounding houses dropped drastically (Mala *et al*. unpublished data). Season affected mosquito counts in breeding sites, confirming previous reports from studies conducted in Mali where malaria transmission during the dry season was found to be undetectable [17]. Rainfall played a minor role in habitat hydrology in Kamarimar but, interestingly, a major one in Tirion. It was noted that larval production significantly peaked in the latter during the wet season when semi-permanent pan dams got filled with rain water. This was accompanied by increased adult catch sizes in adjacent houses (Mala *et al*. unpublished data). Pan dams, which were unique to Tirion, had the highest larval counts but were mainly important during the wet season. Marshes and canals had low larval counts but continued to churn out larvae in dry and wet seasons in both villages. These findings corroborate past findings that showed the most productive habitats per surface area may not necessarily be the most important for spatial and temporal proliferation of vector numbers [18].

*Anopheles gambiae *s.s were not encountered in adult collections, perhaps because the sub-species prefers temporary, sunlit pools [10,11] unlike marshes and drainage canals that were the dominant breeding sites in Baringo. In the rare occasions when temporary habitats were observed after sporadic rain showers, they hardly lasted beyond five days to sustain a complete cycle of larval cohorts to adult stage. These findings are consistent with those of Toure' *et al*. [17] in Mali which showed *Anopheles gambiae *predominated in humid areas; with larval production occurring almost exclusively during rainy periods.

The high catch sizes of *Anopheles. arabiensis *recorded in the present study were expected as these species are known to be more versatile under dry weather conditions than the other sibling species of the *Anopheles gambaie *complex[17]. The nature and seasonal design of habitats also suited their ecology as they are known to exploit permanent, artificial habitats such as rice fields and marshes [19]. Toure' and others [17] noted *Anopheles arabiensis *prevailed in arid areas and likely reproduced throughout the year. Past studies have noted incidences of vectorial complex variation in which certain sibling species dominate during certain times of the year, depending on season. In Tanzania and Nigeria, *Anopheles arabiensis *predominated during the dry season and *Anpheles gambiae*, just after the long rains [19,20]. It would be sensible and logical to conclude from these findings that malaria vectors in semi-arid settings adapt to dry season survival by allowing more hardy sibling species to take up ecological space during the dry season. The availability of permanent water sources complements vector survival by ensuring species that are best adapted to these kinds of habitats such as members of the *Anopheles funestus *group are able to breed and sustain malaria transmission. Large permanent habitats with emergent vegetation are known to favor proliferation of *Anopheles funestus *[10]. These were the main types of breeding sites in Baringo and where *Anopheles funestus *mosquitoes were collected, a pointer to alternative adaptive behavior among vectors based on habitat suitability.

The alternate utilization and quick recolonization of habitats shortly after rainfall in Tirion was interesting. Semi-permanent pan dams in this village were the main drivers of larval production during the wet season while permanent irrigation drainage canals in Kamarimar supported larval breeding during both dry and wet seasons. It is possible that adult mosquitoes carried over from the dry season permanent water sources provided larval "seed" to newly-formed water bodies during the wet season. Further seeding effects could have been provided by other permanent water sources located outside the study villages but from where no sampling was carried out. A good example is found in River Loboi located less than 3 km away from the furthest breeding site in either of the two villages; well within the flight range of gravid *Anopheles gambiae *females [21]. We did not observe any potential obstacles that could have hindered free flight of mosquitoes between the villages and this river, a situation that was favored by an open shrub land that allowed free wind flow.

In various areas with seasonal malaria transmission in Africa, it has been possible to identify local reservoirs of transmission during the dry season [22-24]. Identifying sources of mosquito larvae during the dry season may provide a basis for selective larval control, which may impact on subsequent malaria transmission in the rainy season. The findings of this study provide solid data that can make this dream a reality in Baringo and other semi-arid complexes with similar ecological conditions.

## Conclusion

Breeding of malaria vector mosquitoes in Baringo is driven by predominantly shaded, human-made and permanent breeding sites in which *Anopheles arabiensis *and *Anopheles funestus *breed at low level throughout the year. During the dry season, permanent water sources serve as inocula by providing "larval seed" to freshly formed rain-fed habitats during the rainy season. The highly localized and focal nature of breeding sites in these semi-desert environments provides a good opportunity for targeted larval control since habitats are few, well-defined and easily traceable

## Competing interests

The authors declare that they have no competing interests.

## Authors' contributions

AOM conducted the field studies, analyzed the data and wrote the manuscript. JIS and CMM provided scientific guidance in data collection, analysis and manuscript preparation and planning, and implementation of day-to-day field and laboratory activities. JKN and LWI offered scientific guidance in data analysis and manuscript preparation. EJM provided scientific guidance in data analysis and Manuscript preparation. JIG provided overall supervision of the study and preparation of manuscript. All authors actively contributed to the interpretation of the findings and development of the final manuscript and approved the final manuscript.
